# Cigarette toxin 4-(methylnitrosamino)-1-(3-pyridyl)-1-butanone (NNK) induces experimental pancreatitis through α7 nicotinic acetylcholine receptors (nAChRs) in mice

**DOI:** 10.1371/journal.pone.0197362

**Published:** 2018-06-05

**Authors:** A. A. Alahmari, B. Sreekumar, V. Patel, M. Ashat, M. Alexandre, A. K. Uduman, E. O. Akinbiyi, A. Ceplenski, C. A. Shugrue, T. R. Kolodecik, N. Tashkandi, S. W. Messenger, G. E. Groblewski, F. S. Gorelick, E. C. Thrower

**Affiliations:** 1 Department of Internal Medicine, Section of Digestive Diseases, Yale University School of Medicine, New Haven, CT, United States of America; 2 Veterans Affairs Connecticut Healthcare, West Haven, CT, United States of America; 3 Department of Nutritional Sciences, University of Wisconsin, Madison, Wisconsin, United States of America; 4 Department of Cell Biology, Yale University School of Medicine, New Haven, CT, United States of America; Centro Nacional de Investigaciones Oncologicas, SPAIN

## Abstract

Clinical studies have shown that cigarette smoking is a dose-dependent and independent risk factor for acute pancreatitis. Cigarette smoke contains nicotine which can be converted to the potent receptor ligand and toxin, NNK [4-(methylnitrosamino)-1-(3-pyridyl)-1-butanone]. Previously, we have shown that NNK induces premature activation of pancreatic zymogens in rats, an initiating event in pancreatitis, and this activation is prevented by pharmacologic inhibition of nicotinic acetylcholine receptors (nAChR). In this study, we determined whether NNK mediates pancreatitis through the *α*7 isoform of nAChR using **α**7nAChR knockout mice. PCR analysis confirmed expression of non-neuronal *α*7nAChR in C57BL/6 (WT) mouse and human acinar cells. NNK treatment stimulated trypsinogen activation in acini from WT but not *α*7nAChR^-/-^ mice. NNK also stimulated trypsinogen activation in human acini. To further confirm these findings, WT and *α*7nAChR^-/-^ mice were treated with NNK *in vivo* and markers of pancreatitis were measured. As observed in acini NNK treatment induced trypsinogen activation in WT but not *α*7nAChR^-/-^ mice. NNK also induced other markers of pancreatitis including pancreatic edema, vacuolization and pyknotic nuclei in WT but not *α*7nAChR^-/-^ animals. NNK treatment led to increased neutrophil infiltration, a marker of inflammation, in WT mice and to a significantly lesser extent in *α*7nAChR^-/-^ mice. We also examined downstream targets of *α*7nAChR activation and found that calcium and PKC activation are involved down stream of NNK stimulation of *α*7nAChR. In this study we used genetic deletion of the *α*7nAChR to confirm our previous inhibitor studies that demonstrated NNK stimulates pancreatitis by activating this receptor. Lastly, we demonstrate that NNK can also stimulate zymogen activation in human acinar cells and thus may play a role in human disease.

## Introduction

Acute pancreatitis is an inflammatory disease where up to 30% of patients can develop a severe, often deadly, condition [1]. One of the earliest pancreatitis responses is the premature activation of digestive zymogens in the pancreatic acinar cell. This is followed by inflammation, ischemia, and cell death [1]. Gallstones and alcohol abuse are the most common causes of pancreatitis [2–4]. In addition, cigarette smoke combined with alcohol abuse has long been reported to trigger pancreatitis. Recently, cigarette smoking was also identified as an independent risk factor for initiating acute pancreatitis and a determinant of its severity [4–6]. Many studies have also recognized the independent role of cigarette smoking in chronic pancreatitis [3, 6–10]. However, the mechanism whereby cigarette smoking induces either acute or chronic pancreatitis remains unclear.

Cigarette smoke has numerous potentially toxic components; one of the most harmful and best known is the nitrosated derivative of nicotine, NNK (nicotine-derived nitrosamine ketone or 4-[methylnitrosamino]-1-[3-pyridyl]-1-butanone) [11, 12]. Prior studies in rats demonstrated that NNK causes premature zymogen activation and histological changes comparable to those seen in pancreatitis [1]. In addition, NNK has been shown to enhance the effect of cerulein (CER)-induced pancreatitis, another experimental model of the disease. This indicates that NNK can both initiate pancreatitis and increase disease severity in combination with other agents that cause the disease. Further, a nicotinic acetylcholine receptor was identified as a potential target through which NNK mediates its responses [1]. Originally, nicotinic acetylcholine receptors were identified within the human nervous system [12] and were subsequently identified in rats and mice [13, 14]. We reported that the nicotinic antagonist, mecamylamine, can block NNK-induced zymogen activation in rats [1]. NNK is known to have a high affinity for the α7nAChR with an EC50 in the low nano-molar range [12, 15]. To confirm our findings with the inhibitor, we have used a mouse with genetic deletion of the **α**7nAChR receptor. This animal had no overt phenotype, but exhibited reduced pancreatitis responses when given NNK. These findings confirm that NNK acts, at least in part, on the α7nAChR to induce acute pancreatitis in the mouse.

## Methods and materials

All experiments and procedures using animals were approved by the Veterans Affairs Institutional Animal Care and Use Committee (West Haven, CT). All reagents were purchased from Sigma-Aldrich Biochemical (St. Louis, MO) unless otherwise noted.

### Animal housing and α7nAChR knockout animals

All animals were house under the following conditions: Light/ dark cycle of 12 hours, temperature of 72f +/- 2 degrees with a relative humidity of 30–79%. The α7nAChR Knock out animals were whole body knock-outs and were breed using heterozygous breeding pairs. Weanlings were genotyped and only homozygotes used for experiments.

### Polymerase chain reaction (PCR)

Total RNA was isolated from mouse brain and pancreatic acini using the RNAeasy Midi kit (Qiagen, Valencia, CA) and cDNA was synthesized using the iScript^TM^ cDNA Synthesis kit (Bio-Rad Laboratories, Inc, USA) using random hexamers. PCR was carried out using 2μl first-strand cDNA in a 50μl reaction volume containing [1× PCR buffer–Mg, 1.5 mM MgCl_2_, dNTP mix (0.2 mM each dNTP), 0.2 **μ**M each primer (forward and reverse), and 2U/rxn of Platinum^R^ *Taq* DNA polymerase (Invitrogen, Carlsbad, CA)]. The specifics for both mouse and human primers and amplification conditions are as follows. Mouse primers used were based on those previously used in rat as the sequence in question is the same, *α7*nAChR F: 5’-ATCTGGGCATTGCCAGTATC-3’, R: 5’-TCCCATGAGATCCCATTCTC-3’ [1, 16]. Amplification conditions were initial denaturation (3 min, 94°C) then 45 cycles of denaturation (94°C, 45 sec), annealing (49°C, 30 sec), and extension (72°C, 30 sec). For human PCR the above primers were modified to correspond to the human sequence *α7*nAChR F: 5’-TTCTGGGCATTGCCAGTACC-3’, R: 5’-TCCCACAGGTCCCATTCTC and the amplification conditions used were the same as used above for mouse except that the annealing step which was modified to (51°C, 30 sec). PCR products were analyzed on 1× TAE agarose gel that contained ethidium bromide.

### Acinar cell preparation

Acinar cells were isolated as previously described [2]. Briefly, mice or rats (Charles River Laboratories, Wilmington, MA) were euthanized by CO_2_ inhalation. The pancreas was minced in buffer-A [10 mM HEPES (pH 7.4), 95 mM NaCl, 4.7 mM KCl, 0.6 mM MgCl_2_, 1mM NaH_2_PO_4_, 10mM glucose, 2mM glutamine, 0.1% bovine serum albumin, and 1× MEM amino acids (GIBCO-BRL, San Jose, CA)] and washed three times. Cells were then digested for 1h at 37°C in buffer-A containing 50 U/ml of type IV collagenase (Worthington, Freehold, NJ) with sustained shaking. The digest was filtered through a 200 μm mesh (Sefar American, Depew, NY), and the resulting groups of acinar cells were distributed in a 24-well Falcon tissue culture plate (Becton Dickinson, Franklin Lakes, NJ) and placed in a water bath shaking at 90-rpm under constant oxygen flow to recover. Human pancreatic acinar cells were prepared as previously described[17].

### In-vitro pancreatic acinar cell treatment with NNK and/or CER

After isolation acini were recovered for 2 hours. After recovery acini were treated with PBS 90 min (unstimulated control), NNK 10 or 100nM (Toronto Research Chemicals, Toronto, ON, Canada or Sigma-Aldrich Biochemical, St. Louis, MO) for 90 min, PBS for 60 min followed by 100 nM CER for 30 min, or 100 nM NNK for 60 min followed by 100 nM CER for 30 min.

### In-vitro pancreatic acinar cell experiments to examine the role of calcium and PKC activation

Acinar cells were prepared as above from rats. To examine the role of extracellular calcium, acini were pretreated with eBAPTA-AM (50**μ**M) for 30 min. Then washed into either calcium free or calcium containing buffer and immediately stimulated [18] with NNK (100nM / 90min). If inhibition of PKC was to be examined acini were pre-treated with the PKC inhibitor GF-109203X (10**μ**M) for 120min before the addition of NNK (100nM / 90min) [19, 20].

At the end of the incubations acini were collected for determination of zymogen activation and total amylase. All samples are frozen at -80°C until assayed.

### In vivo cerulein model of pancreatitis

NNK was diluted in sterile Phosphate-buffered saline (PBS). Acute pancreatitis was induced by giving mice 6 hourly intra-peritoneal (IP) injections in a total volume of 200μl as follows: 1) sterile PBS, 2) NNK (100 mg/Kg body weight), 3) CER (40 μg/Kg body weight) or 4) a combination of NNK+CER. Animals were anesthetized, blood was collected by exsanguination and assayed for serum amylase. Each pancreas was harvested and analyzed for zymogen activation, histological changes, and immune responses.

### Enzymatic activity assays

Trypsinogen activation assays were performed as a marker of zymogen activation [1]. Briefly, previously frozen samples from *in vitro* and *in vivo* studies were thawed, homogenized in trypsin assay buffer at a ratio of 20 ml of buffer per gram tissue, and centrifuged at 500g for 10 minutes to generate a postnuclear supernatant. Samples were assayed in a 24-well culture plate (Greiner Bio-one Cellstar TC-Plate), each well was loaded with: 1) 100μl of postnuclear supernatant; 2) 350 μl of zymogen assay buffer [50 mM Tris (pH 8.1), 150 mM NaCl, 1 mM CaCl2] and the assay was started by addition of 50 μl of 400 μM enzyme substrate (40 μM final) [fluorometric trypsin substrate (catalog no. 3135-v, Peptides International, Louisville, KY). The plate was read with a fluorometric microtiter plate reader (model HTS 7000, Perkin-Elmer Analytical Instruments, Shelton, CT; 380-nm excitation, 440-nm emission, 20 reads/10 min). And slopes corresponding to enzymatic activity determined.

### Histology and immunohistochemistry

Pancreatic tissues from in vivo studies (1×1 mm) were fixed in 10% formalin. Tissue was processed by Yale Pathology. Samples were dehydrated, embedded in paraffin, and sectioned (5 **μ**m) followed by staining with hematoxylin and eosin (H&E) or immunostaining for neutrophils (Ly-6G). H&E slides were reviewed and ranked for the amount of edema, number of pyknotic nuclei, and vacuoles using an Axiophot microscope (Carl Zeiss, Thornwood, NY) at ×40 magnification, and images were collected with a Spot Digital camera (Diagnostic Instruments, Sterling Heights, MI). Tissue sections were scored in a blinded manner using a histological scoring system [21]. For immune responses, slides were assessed by counting dark brown staining cells (Neutrophils). All slides were assessed and scored in a blinded manner.

### Statistical analysis

Data represents the mean values ± Standard Error of the Mean using a minimum of three individual experiments, with each condition replicated. Statistical significance was determined by T-test (in-vitro) or the Mann-Whitney test for comparing ranks (in-vivo). Statistical significance was set at P < 0.05.

## Results

### Presence of α7nAChR on mouse pancreatic acini was confirmed by PCR analysis

**Fig 1:**
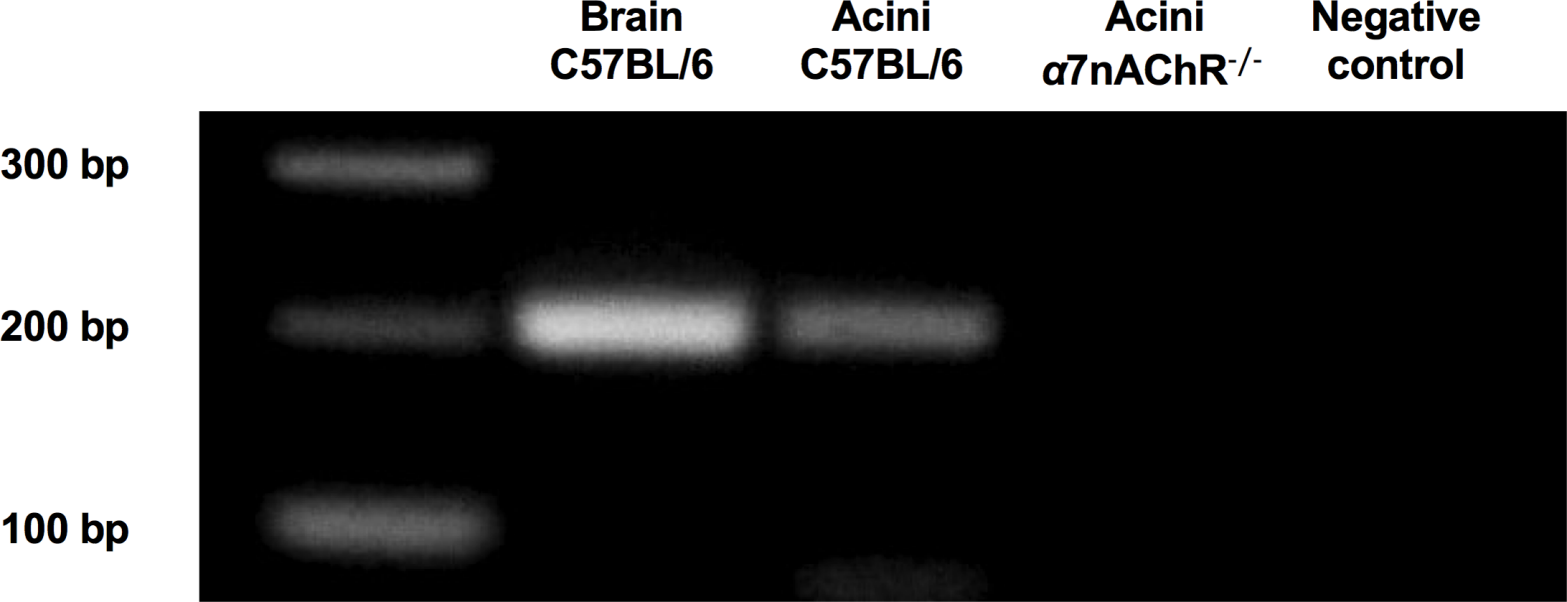
The nonneuronal *α*7-nicotinic ACh receptor (*α*7nAChR) is expressed in acinar cells of WT mice and humans but not those of *α*7nAChR^-/-^ mice. cDNA from unstimulated tissues was used to determine the presence and absence of the *α*7nAChR on mouse (wild type and knockout) acinar cells. Positive control was brain; negative control reactions contained no cDNA.

We have previously shown that the *α*7nAChR is present on rat pancreatic acinar cells and that inhibition of this receptor abrogates NNK-mediated zymogen activation in rats [1]. In the current study, PCR was performed to confirm the presence of the *α*7nAChR in C57BL/6 mouse (wild type, WT) acini. RNA from brain was used as a positive control. PCR analysis showed a band of the expected size (199 nt) for the *α*7nAChR in brain tissue and acinar cells isolated from WT mice (Fig 1). No PCR product was amplified from cDNA from *α*7nAChR^-/-^ acini, confirming the deletion of this receptor (Fig 1).

### NNK induces trypsinogen activation via α7nAChRs in isolated pancreatic acini

**Fig 2:**
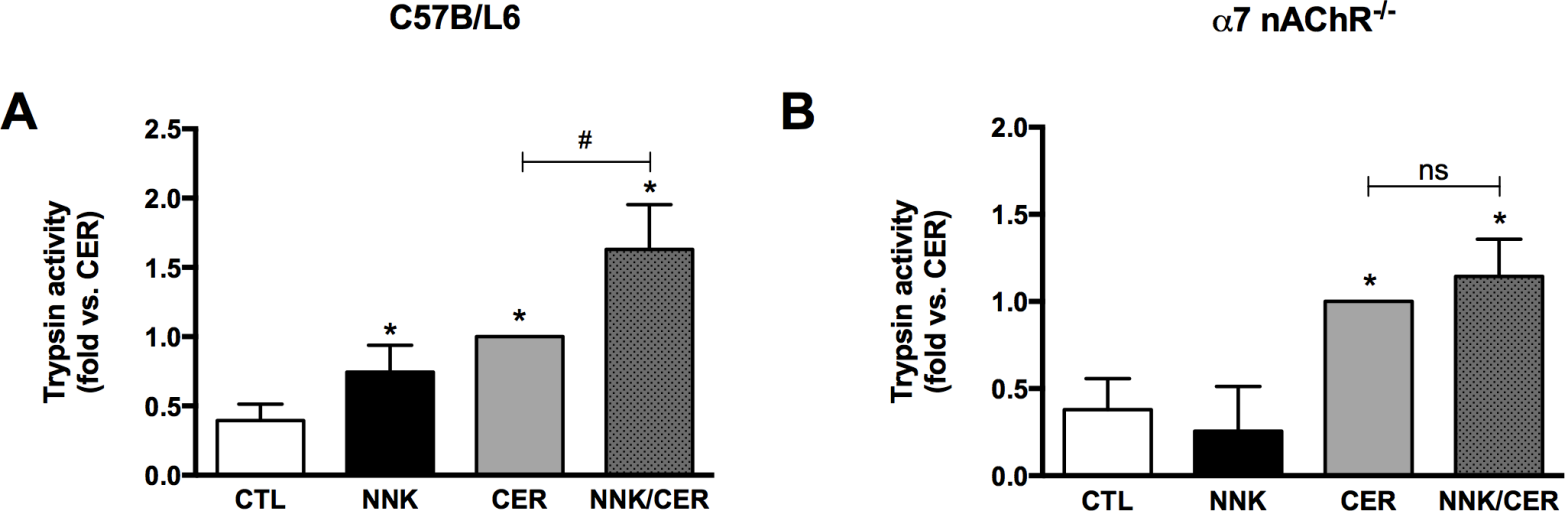
NNK stimulation of trypsinogen activation is receptor-dependent in vitro. **(A)** NNK induces zymogen activation in pancreatic acinar cells isolated from WT mice, **(B)** but not in *α*7nAChR^-/-^ isolated acini. Acinar cells were treated with NNK (100 nM), CER (100 nM), and combination of both. NNK (100 nM) alone induced trypsin activity, and NNK+CER further enhanced trypsin activity compared to those induced by CER (100 nM) alone. Values are means ± SE; n = 6. *P < 0.05 vs. CTL. #P < 0.05 vs. CER. ns = not significant.

NNK treatment caused increased trypsinogen (zymogen) activation in WT acini compared to controls (Fig 2*A*). In contrast, there was no significant effect of NNK on zymogen activation in acini from *α*7nAChR^-/-^ mice (Fig 2*B*). When combined with CER, NNK showed an additive effect on trypsinogen activation in WT mice (Fig 2*A*); no significant increase was noticed in *α*7nAChR^-/-^ mice (Fig 2*B*). These findings suggest that in acini NNK induces zymogen activation through an *α*7nAChR-mediated pathway.

### NNK induces trypsinogen activation through α7nAChRs in vivo

**Fig 3:**
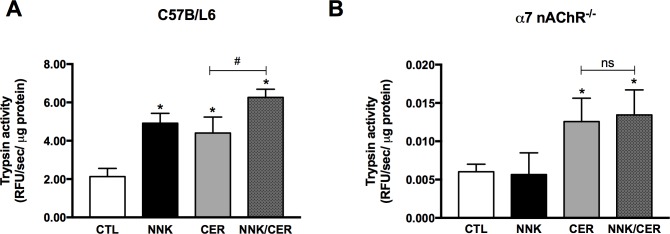
NNK stimulation of trypsinogen activation is receptor-dependent in vivo. **(A)** NNK induces zymogen activation in vivo within WT mice, **(B)** but not in *α*7nAChR^-/-^ mice. Mice were injected with NNK (100 mg/kg), CER (40 **μ**g/kg), or a combination of both. **(A)** NNK (100 mg/kg) alone induced trypsin activity, and NNK+CER further enhanced trypsin activity compared to those induced by CER alone. **(B)** NNK effect on *α*7nAChR^-/-^ mice is abrogated. Values are means ± SE; n = 6. *P < 0.05 vs. CTL. #P < 0.05 vs. CER. ns = not significant.

To confirm our in-vitro results in an animal model WT and *α*7nAChR^-/-^ mice were injected with PBS, CER, NNK or the combination of CER/NNK. A significant increase in trypsinogen activation was seen in WT mice treated with NNK compared to PBS control (Fig 3*A*). On the other hand, NNK did not stimulate trypsinogen activation in *α*7nAChR^-/-^ mice (Fig 3*B*). CER significantly increased trypsinogen activation in both WT and *α*7nAChR^-/-^ mice demonstrating that deletion of this receptor does not impair activation by other mechanisms (Fig 3*A and 3B*). CER stimulated trypsinogen activation was enhanced by NNK treatment in WT mice (Fig 3*A*) but not in *α*7nAChR^-/-^ mice (Fig 3*B*). Together, this data confirms the in-vitro results demonstrating that NNK mediates zymogen activation through the *α*7nAChR.

### NNK induces trypsinogen activation through α7nAChRs in human acinar cells

**Fig 4:**
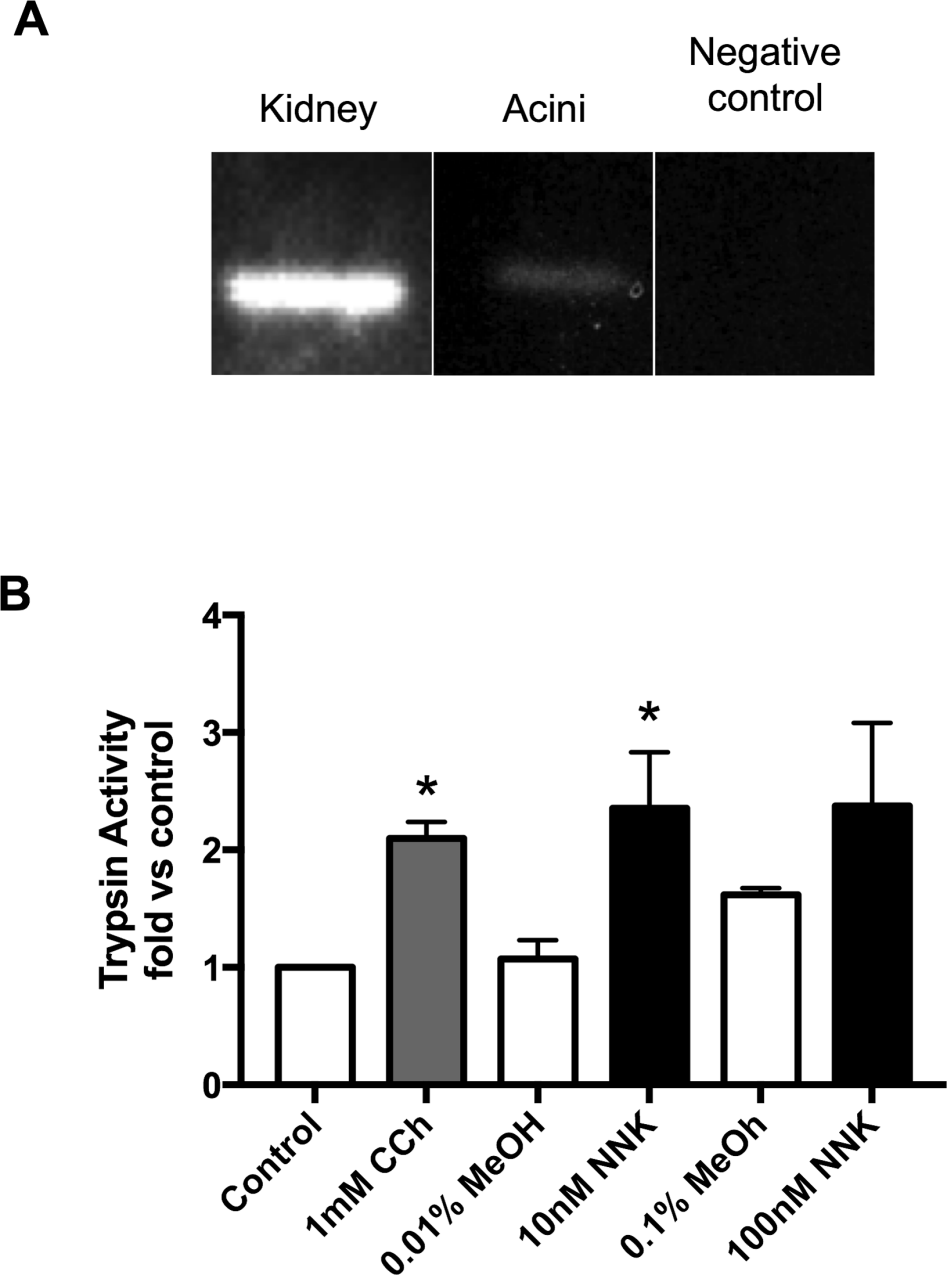
NNK stimulates trypsinogen activation in human acinar cells. (**A):** cDNA from human acinar was used to determine the presence or absence of the *α*7nAChR. Brain was used as a positive control and the negative control contained no cDNA. **(B):** Human Acinar cells were incubated with either carbachol (CCh 1mM, 30 min), NNK (10 or 100 nM) or methanol 0.01–0.1% (respectively) as methanol was used to solubilize NNK.

We next determined if human cells express the *α*7nAChR and whether they respond to NNK stimulation with trypsinogen activation. Using primers specific for human *α*7nAChR, we detected the receptor in both the positive control (kidney) as well as in pancreatic acinar cells, but not in the negative control (Fig 4*A*). Because it is controversial whether or not human acinar cells have functional CCK receptors [22, 23] we used carbachol, a muscarinic receptor agonist, as a control to test for human acinar cell responsiveness. We found that NNK (10 nM) significantly stimulated trypsinogen activation above the methanol (MeOH 0.01%) control. Trypsinogen activation with 100 nM NNK (MeOH 0.1%) was similar to that seen with 10 nM but was not significant due to the higher baseline seen with 0.1% MeOH (Fig 4*B*). These studies shows that both murine and human acinar cells express the *α*7nAChR and respond to NNK with trypsinogen activation.

### NNK induces histological changes associated with pancreatitis in wild type but not α7nAChR^-/-^ mice

**Fig 5:**
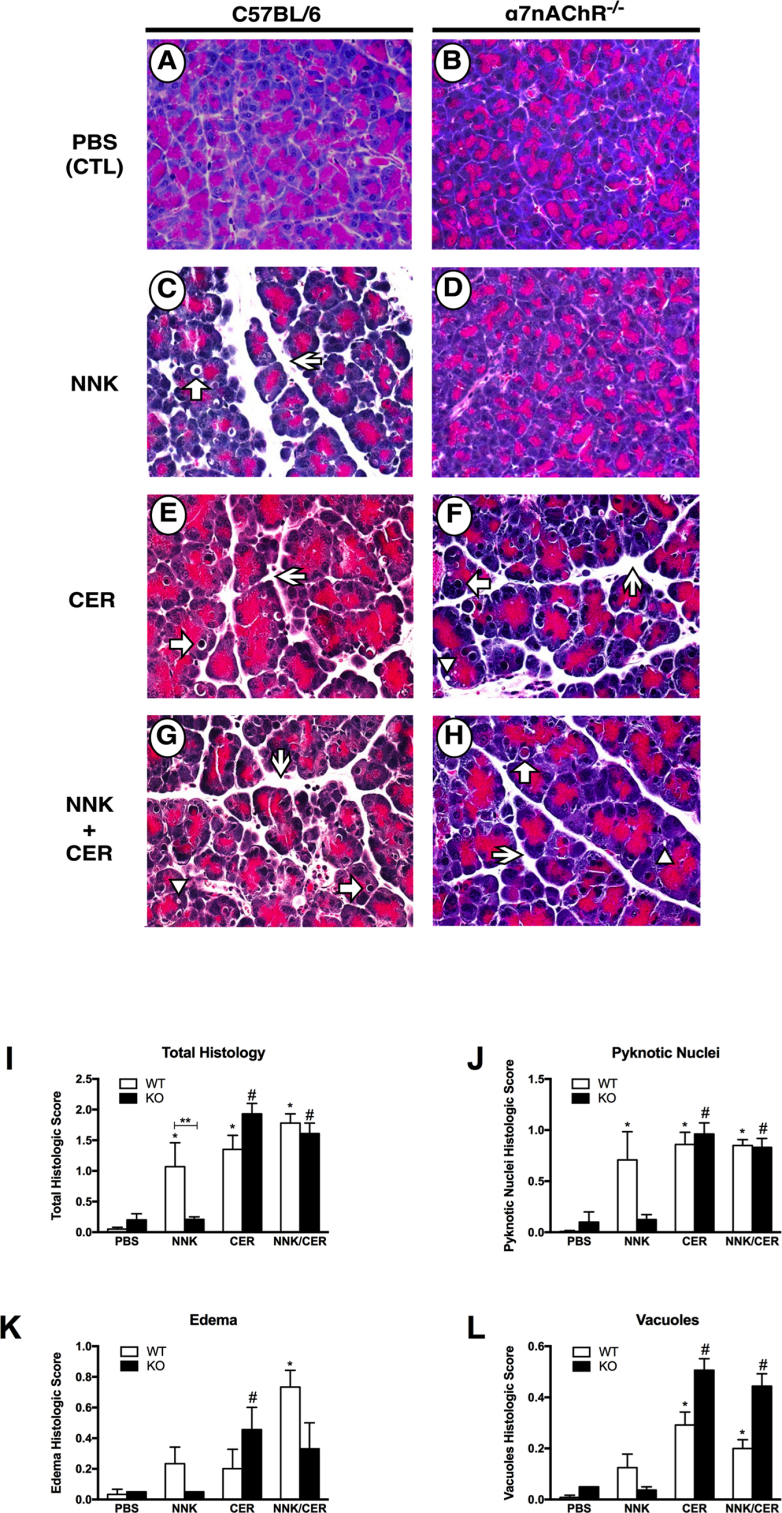
NNK-mediated histological changes in vivo are receptor-dependent. **(A & B):** no histological changes in PBS-treated pancreatic tissues. (**C & D)**: NNK caused histological changes in WT, but not in *α*7nAChR^-/-^ mice. **(E—H)**: Histological changes are observed in tissues treated with CER alone or in combination with NNK. Arrows = pyknotic nuclei; Arrowheads = Vacuoles; Sharp arrows = edema. **(I)**: total histological score of all parameters. **(J, K, and L)**: categorized scores for pyknotic nuclei, edema, and vacuoles, respectively. *P < 0.05 vs. WT-PBS. #P < 0.05 vs. KO-PBS. **P < 0.05 vs. KO-NNK.

After in vivo treatment, histological markers of pancreatitis were evaluated and scored in a blinded manner. There was no difference between WT and *α*7nAChR^-/-^ mice in PBS controls (Fig 5*A and 5B*). In WT animals NNK (Fig 5*C*), CER (Fig 5*E*) and the combination of both (Fig 5*G*) showed significant changes in pancreatic histology. In *α*7nAChR^-/-^ mice treated with CER (Fig 5*F*) there was no difference compared to WT CER mice (Fig 5*E*). When *α*7nAChR^-/-^ mice were treated with NNK (Fig 5*D*) there were no histologic changes and tissues appeared similar to PBS controls (Fig 5*A and 5B*). When *α*7nAChR^-/-^ mice were treated with the combination of CER+NNK (Fig 5*H*) the histology appeared similar to that of CER treatment alone (Fig 5*E and 5F*). When scores were quantified (Fig 5*I–5L*) there was a significant increase in total histologic score associated with CER, NNK and the combination in WT mice as well as *α*7nAChR^-/-^ mice treated with either CER alone or in combination with NNK (Fig 5*I*). Consistent with NNK working through activation of the *α*7nAChR, knockout mice treated with NNK alone showed no significant increase in total histologic score compared to WT control (Fig 5*I*). The apparent increase in pyknotic nuclei (Fig 5*J*), edema (Fig 5*K*) and vacuolization (Fig 5*L*) seen with NNK treatment in WT mice was absent in *α*7nAChR^-/-^ mice (Fig 5*J–5L*). Although there appeared to be changes in these same parameters (i.e. pyknotic nuclei, edema, and vacuolization) in the CER and CER+NNK treated animals, these differences were not significant (Fig 5*I–5L*). These data suggest that in addition to zymogen activation, histologic damage caused by NNK *in-vivo* likely requires activation of the *α*7nAChR. Further, the effects of CER in the KO are complex and suggest distinct roles for this signaling pathway in mediating various parameters of pancreatitis.

### Pancreatic neutrophil infiltration during acute pancreatitis is α7nAChR dependent

**Fig 6:**
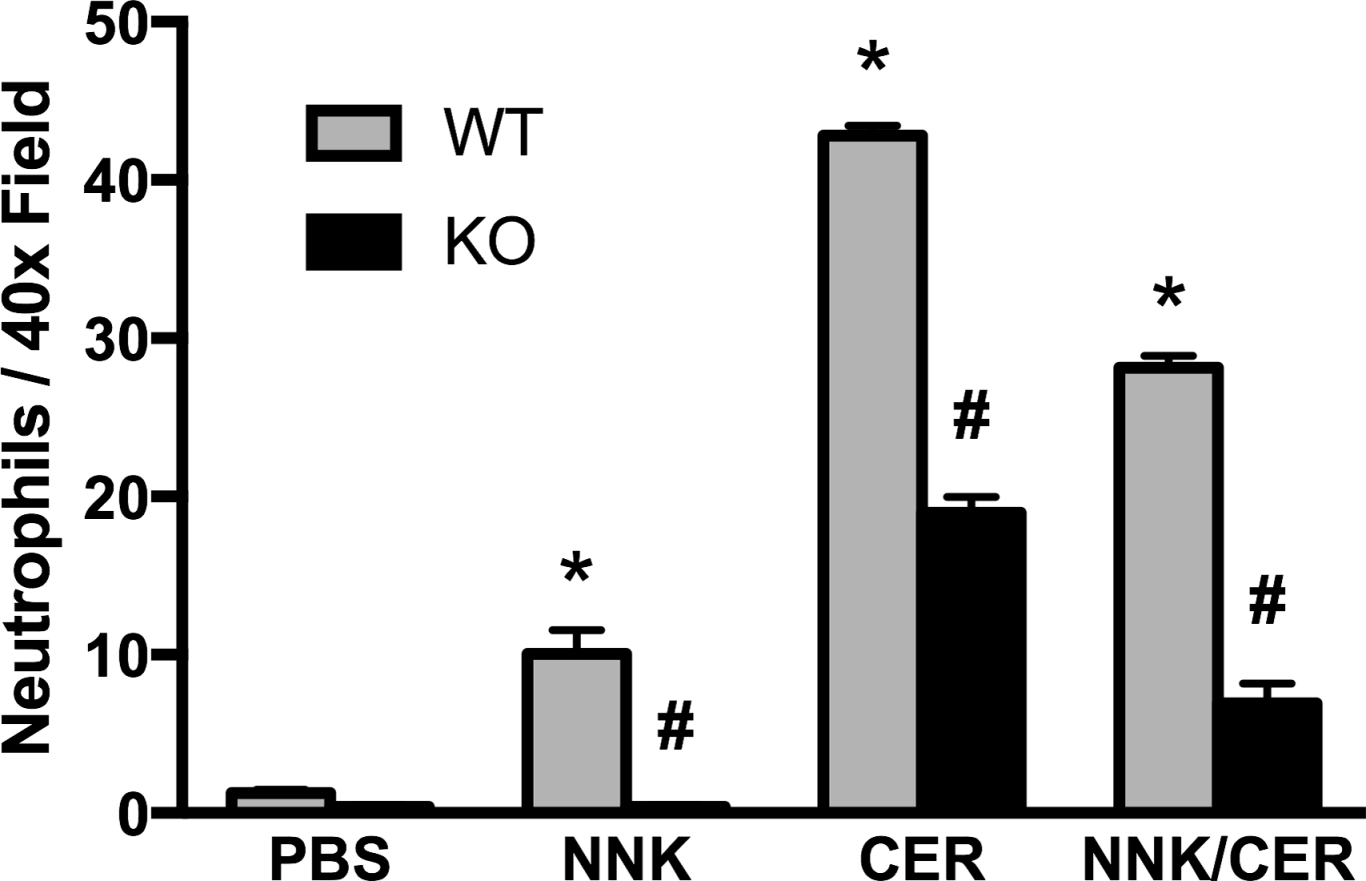
Pancreatic neutrophil infiltration during acute pancreatitis is receptor dependent. NNK caused neutrophil infiltration in WT but not *α*7nAChR^-/-^ mice. Also, knocking out *α*7nAChR decreases neutrophils migration into CER-treated pancreatic tissues, and this effect was more intense when NNK was given in combination with CER. Studies in wild type and *α*7nAChR^-/-^ (KO) mice. Values are means ± SE; n ≥ 4. *P < 0.05 vs. PBS WT. #P < 0.05 vs. analogous WT.

When neutrophil infiltration was examined as a marker of inflammation few neutrophils were observed in PBS-treated mouse pancreas (WT and *α*7nAChR^-/-^, Fig 6). In WT mice, NNK, CER, and the combination of both resulted in significant increases in neutrophil infiltration. There was also a significant increase in infiltration in CER and CER+NNK treated *α*7nAChR^-/-^ animals. However, CER, NNK and the combination had significantly fewer neutrophils in the *α*7nAChR^-/-^ pancreas compared to same treatments in WT mice. There was also less infiltration in animals treated with CER+NNK than CER alone (Fig 6). These studies suggested that the *α*7nAChR could be a negative regulator of neutrophil inflammation.

### Downstream targets of α 7nAChR activation

**Fig 7:**
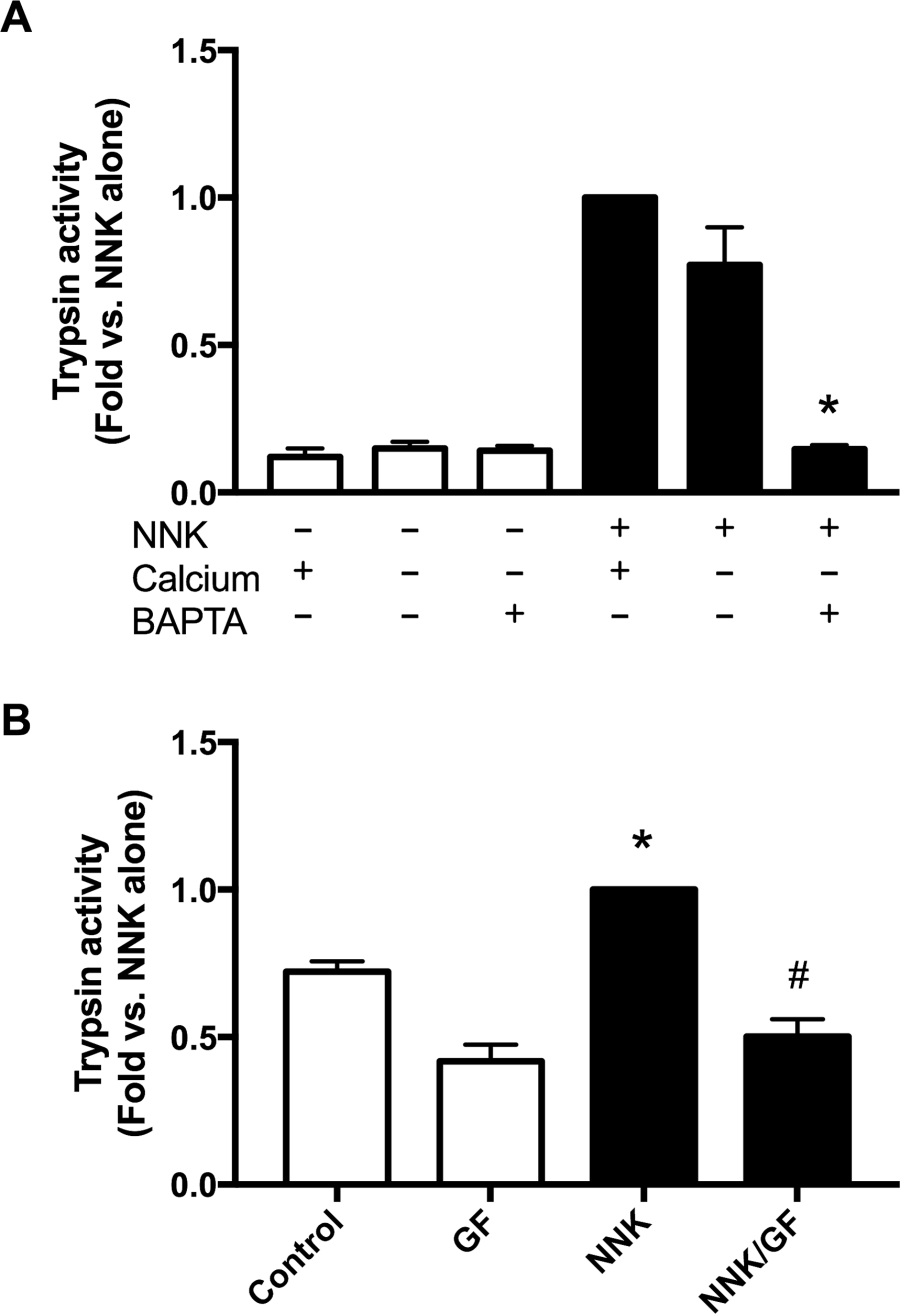
Depletion of intracellular calcium or inhibition of PKC blocks NNK induced trypsinogen activation. **(A)** Trypsinogen activation by NNK (100 nM) was not inhibited in calcium free media. But, preincubation with the membrane permeable calcium chelator BAPTA-AM (10 **μ**M, 30 min) followed by switching to a calcium free media significantly inhibited trypsinogen activation. **(B)** NNK induced trypsinogen activation was inhibited by preincubation with the broad-spectrum PCK inhibitor GF-109203X (10 **μ**M) for 120 min. Values are means ± SE; n = 3. *P < 0.05 vs. control, #P < 0.05 vs. NNK alone.

Calcium signaling and PKC activation, two downstream targets of α7nAChR activation are known to be involved in secretagogue stimulated zymogen activation. Here we examined whether these mechanisms are involved in NNK induced zymogen activation. When acini in calcium free media were exposed to NNK there was a reduction of trypsinogen activation, but it was not significant (Fig 7*A*). However when acini were pretreated with the membrane permeable calcium chelator BAPTA-AM and then switched to calcium free media, NNK stimulated trypsinogen activation was inhibited back to baseline (Fig 7*A*). Similarly, PKC inhibition using pre-incubation with the broad-spectrum PKC inhibitor GF-109203X significantly inhibited NNK stimulated trypsinogen activation (Fig 7*B*).

## Discussion

Studies have found a direct link between cigarette smoke and pancreatitis and have defined smoking as an independent risk factor for pancreatic disease [3, 6]. A previous study in rats has shown that the nicotine metabolite NNK, a potent tobacco carcinogen, can cause and enhance secretagogue-stimulated acute pancreatitis [1]. This study also ruled out both the cholecystokinin and β1/β2 adrenergic receptors as acinar cell targets for NNK [1]. Nicotinic receptors were determined to be the most likely receptor target on the pancreatic acinar cell. This was based on experiments using mecamylamine, a non-isoform specific inhibitor of nicotinic receptors and the presence of the α7nAChR on the pancreatic acinar cell [1]. To confirm and expand our studies in rats, here we used to a genetic approach in mice with whole body deletion of the α-7 nAChR to demonstrate that that the molecular target of NNK is this nicotinic receptor [24].

The concentrations of NNK used *in-vivo* (100 mg/kg) in this study are the same as used in studies to induce lung tumorigenesis in mice [25, 26]. Although, The amount of NNK used *in-vitro* (100 nM) is within the range found in the pancreatic juice of smokers (1.37–604 ng/ml or approximately 7 nM-3 **μ**M) [27] we are likely modeling the extremes of what may be see in humans. One issue with modeling human disease in rodents is that disease progression in humans takes place over many years a condition we are not able to reproduce in animal models. Keeping this in mind we found that this treatment regime had a similar effect on pancreatitis responses in mouse as was previously found in rat [1].

When we examined the effects of NNK *in vitro*, comparing acinar cells isolated from WT and *α*7nAChR^-/-^ mice we found that NNK did not cause trypsinogen activation nor did it enhance CER-stimulated zymogen activation in *α*7nAChR^-/-^ mice (Fig 2). We then examined the effects of NNK in an *in-vivo* model of pancreatitis and found a similar effect of NNK in the *α*7nAChR^-/-^ mice (Fig 3). Unexpectedly, we observed a decrease in basal and CER-stimulated trypsin activity in the *α*7nAChR^-/-^ mice. Though outside the scope of this paper, there may several reasons for this. We have preliminary data using enterokinase to activate trypsinogen in pancreatic homogenates that *α*7nAChR^-/-^ mice have less activatable trypsinogen than WT mice (data not shown). However, reduced zymogen levels on their own are probably not sufficient to account for the decreased activation seen in the KO mice. Alternately, trypsinogen activation requires the presence of active lysosomal enzymes, cathepsin B in particular, and a low pH compartment [28, 29]. If the *α*7nAChR were involved in either lysosomal activation/processing or compartmental acidification its loss could lead to the lower basal and stimulated trypsinogen activation seen in *α*7nAChR^-/-^ mice.

In addition to trypsinogen activation, we examined histologic parameters of pancreatitis and neutrophil infiltration in WT and *α*7nAChR^-/-^ animals. As observed in rats [1], NNK treatment in WT animals resulted in histologic changes similar to those seen in CER-treated animals. The combination of both showed varying degrees of additivity. In the *α*7nAChR^-/-^ animals, there was no increase in any histologic parameter of pancreatitis with NNK treatment. This is consistent with NNK effects being mediated by *α*7nAChR. Unexpectedly, CER treatment in the *α*7nAChR^-/-^ animals resulted in significant changes in histologic parameters of pancreatitis including increased edema and vacuoles, a worse overall histologic score, but reduced neutrophilic infiltration. Though investigating these responses is beyond the scope of our present work, it is possible that the *α*7nAChR could mediate CER-dependent responses.

It has been shown that both neutrophils and macrophages express α7nAChRs [30, 31]. The effects of NNK on inflammation can be complex. In alveolar macrophages NNK inhibits the production of pro-inflammatory mediators and increases the production of anti-inflammatory mediators resulting in an immunosuppressive environment in the lung [32]. It has also been shown that neuronal derived acetylcholine can activate the α7nAChR on macrophages resulting in the selective inhibition of pro-inflammatory cytokine production while having no effect on the production of anti-inflammatory cytokines resulting in a net anti-inflammatory response[33]. In contrast, in the liver NNK treatment causes an increase in pro-inflammatory cytokines [34]. When we examined neutrophil infiltration in our mouse model, we found that in WT mice both CER and NNK caused a significant increase in neutrophil infiltration. Interestingly, when WT animals were treated with both CER and NNK there were fewer neutrophils observed than in CER alone suggesting that NNK may be having at least a partial anti-inflammatory effect. This is consistent with the anti-inflammatory effect seen when the α7nAChR on macrophages is activated with nicotine [35]. Therefore, NNK could be activating the *α*7nAChR on neutrophils thus preventing their infiltration. This explanation, however is problematic when viewing the comparable data from the *α*7nAChR^-/-^ mice; there is no neutrophil infiltration with NNK alone, CER-induced neutrophil infiltration is reduced, and NNK/CER induced infiltration is reduced versus WT. This would indicate that activation of the *α*7nAChR is not mediating an anti-inflammatory effect but a pro-inflammatory one. This is bolstered by the reduced neutrophil infiltration seen with CER stimulation in *α*7nAChR^-/-^ mice. Taken together the NNK-mediated inflammatory response in pancreatitis and the involvement of the *α*7nAChR is complex and clearly requires further studies into the involvement of α7nAChR activation on acinar cells using a targeted deletion model.

Our findings reveal that NNK causes trypsinogen activation and histological changes, with limited leukocyte infiltration, through *α*7-nAChR. We also examined potential downstream targets of *α*7nAChR activation. One possible downstream pathway is intracellular calcium signaling. The *α*7nAChR has a high permeability to calcium [36], and its activation causes cytoplasmic calcium levels to rise [37]. In pancreatic acinar cells increased intracellular calcium accompanies premature zymogen activation [38, 39]. In this study we found that NNK-stimulated zymogen activation is reduced when acini were incubated in calcium-free media along with chelation of intracellular calcium. This suggests that intracellular calcium stores and their release of calcium have important roles in NNK-stimulated zymogen activation.

Activation of the *α*7nAChR is also known to activate Protein kinase-C (PKC) [40, 41]. In pancreatic acinar cells the inhibition or deletion of different PKC isoforms can lead to the inhibition of CER-stimulated zymogen activation [19, 20]. In this study we used the broad-spectrum PKC inhibitor GF109203X to examine the role of PKC in NNK-induced trypsin activation. We found that inhibition of PKC reduced NNK-stimulated trypsin.

Another possible downstream pathway that has been investigated is thiamine (vitamin B_1_) uptake by pancreatic acinar cells [42–44]. NKK has been shown to inhibit thiamine uptake in pancreatic acinar cells [42]. Once in the cytosol, thiamine is converted to thiamine pyrophosphate (TPP), a derivative crucial to normal mitochondrial function. TPP is transported into the mitochondria via the mitochondrial TPP transporter (MTPPT), a product of the SLC25A19 gene. In a pancreas cell line (266–6) transport of TPP into the mitochondria as well as MTPPT protein levels are reduced when chronically treated with NNK. [43] This reduction is blocked by the *α*7nAChR antagonist mecamylamine but not by inhibition of the beta-adrenergic receptor. In addition, NNK treatment had no effect on levels of MTPPT protein or SLC25A19 mRNA expression in *α*7nAChR^-/-^ mice [43]. This suggests that NNK could induce mitochondrial dysfunction by lowering TPP uptake resulting in decreased levels of ATP making cells more sensitive to oxidative injury and cell death. However, there are differences in both time of exposure and concentration of NNK used to investigate thiamine uptake and our model. In the thiamine uptake model 266–6 cells are exposed to 3 μM NNK for 24 hours, whereas in this study freshly isolated acini are exposed to 100 nM NNK for only 90 min. *In vivo* the same concentration of NNK was used (100 mg/Kg body weight) in both models, but the time course of administration was different. In the thiamine uptake model NNK was given 3x/week IP for two weeks whereas in our study 6 hourly IP injections were given and mice were euthanized 1 hour after the last injection. Despite these differences, the thiamine uptake studies suggest that mitochondrial function and ATP levels could also be affected in our short-term model.

Though our studies have shown a role for the α7nAChR in transducing the effects of the cigarette toxin NNK, there are limitations to this model of pancreatitis. This study only examined early stages of mild acute pancreatitis and does not address how the chronic administration of NNK could affect the α7nAChR.

This paper examines neutrophil infiltration but did not look at macrophages which have been shown to mediate an anti-inflammatory effect through α7nAChR when given nicotine [35]. In addition, nicotine was found to reduce the severity of acute pancreatitis by controlling CD4^+^CD25^+^ regulatory T cells (Tregs) [45]. These cells were found to express α7nAChR and were shown to have a suppressive capacity when stimulated with nicotine [46]. This suppressive response was reversed using a selective α7nAChR antagonist, which suggests a key role of Tregs in mediating an anti-inflammatory effect [46].

Lastly, this study used NNK, one of the more than 5000 compounds found in cigarette smoke [47]. NNK was chosen as it is one of the most toxic components of cigarette smoke but the effects of other toxic constituents of cigarette smoke alone or in combination with NNK may also lead to pancreatitis. Therefore, a model of cigarette smoking would be useful to confirm these outcomes.

In summary, our data show that the nicotine metabolite NNK, a potent toxic component of cigarette smoke, causes trypsinogen activation and cellular damage leading to pancreatitis, and that these effects are mediated through the non-neural α7-nAChR pathway. Furthermore, we have shown that changes in intracellular calcium and activation of PKC, both downstream targets of *α*7nAChR activation, are involved in NNK-induced pancreatitis. This study forms the basis for future research examining the effects of long term treatment with NNK or cigarette smoke and the involvement of the *α*7nAChR.
